# A Pilot Study Examining Biofeedback and Structured Napping to Promote Medical Student Wellbeing

**DOI:** 10.15694/mep.2019.000110.1

**Published:** 2019-05-27

**Authors:** Caridad Hernandez, Katherine Daly, Anuja Mehta, Marcia Verduin

**Affiliations:** 1 University of Central Florida College of Medicine

**Keywords:** medical students, well-being, biofeedback, structured napping

## Abstract

This article was migrated. The article was marked as recommended.

**Objective:** To examine medical students’ engagement in wellness activities and evaluate the effects of biofeedback and structured napping on measures of stress, burnout and wellbeing.

**Method:** A randomized trial of heart-rate variability (HRV) biofeedback and structured napping used by pre-clinical medical students at the University of Central Florida College of Medicine compared with a control group was conducted. Baseline measurement occurred in August 2016 with the follow-up period in March 2017.

To measure biofeedback, participants used Heartmath Biofeedback® with Inner Balance® software to record HRV measurements while they engaged in self-guided breathing three times weekly. The biofeedback device connected to participants’ iPhone or iPad with a sensor that clipped to users’ earlobes. HRV recordings were stored in a heart-cloud database, and participants had the option to share their recordings with the researchers. Participants used sleep pods (MetroNaps Energy Pods®) to engage in 20-minute structured naps three times weekly.

Participants completed six psychosocial self-report questionnaires at baseline (T1) and two follow-up points (T2, T3). The questionnaires included the Interpersonal Reactivity Index; Perceived Stress Scale; Quality of life scale; Oldenburg Burnout Inventory; and the Physician Well-Being Index.

**Results:** Forty-two students enrolled in the study. Throughout the study, participants recorded 276 structured naps lasting approximately 20 minutes in duration and shared 24 personalized biofeedback recordings.

**Conclusions:** Promotion of structured napping offers promise as an institution-initiated wellness intervention to promote medical students’ mental health and wellbeing. HRV biofeedback warrants further study given the lack of conclusive findings in this study.

## Introduction

A burgeoning body of literature indicates that students’ wellbeing suffers during medical school and there is a high prevalence of psychological distress ( Dyrbye, et al., 2008, 2011, 2014, MacLean, et al., 2016). Identifying and addressing factors that contribute to students’ distress is critical given the association between burnout and high rates of anxiety, depression, suicidal thoughts, and lapses in professionalism ( Dyrbye *et al.*, 2010) . Studies with medical trainees have identified a direct correlation between diminished mental health, increased fatigue, and decline in empathy for patients ( Bellini, Baime and Shea, 2002; Bellini and Shea, 2005; Dyrbye *et al.*, 2008; Neumann *et al.*, 2011). Recognizing the potential impact on the future physician workforce ( Enoch et al. 2013) and ultimately on patient outcomes ( Fahrenkopf *et al.*, 2008; West *et al.*, 2009), effective strategies are needed to reduce burnout and enhance students’ wellbeing ( AAMC, 2016). This has been recognized by the Liaison Committee on Medical Education (LCME) accreditation requirement for implementing programs that emphasize medical students’ wellbeing and “facilitate their adjustment to the physical and emotional demands of medical education” ( LCME, 2016).

There is evidence to suggest that interventions that promote positive mental health and wellbeing forestall the development of burnout and other negative outcomes ( Dyrbye *et al.*, 2012). Medical schools embarking on institutional wellness initiatives have to contend with the paucity of empirical studies to help select cost-effective options ( Drolet and Rodgers, 2010; Winseman *et al.*, 2015). Studies are needed to build evidence for adopting specific practices that include explanatory theoretical frameworks in order to facilitate the exploration of novel approaches (Holmes *et al.*, 2017).

Mind-body strategies have emerged as a promising approach to reduce medical students’ distress and promote wellbeing ( Shapiro, Schwartz and Bonner, 1998; Rosenzweig *et al.*, 2003). Mindfulness-based stress reduction (MBSR) has been shown to reduce psychological distress and increase positive mental health ( van Dijk *et al.*, 2017). Mind-body skills training programs (including biofeedback, guided imagery, relaxation and meditation) have demonstrated a modest reduction in distress and improved ability to respond to stress ( Kraemer *et al.*, 2016). The health benefits derived from mindfulness practices may be mediated, in part, by improved emotion regulation, thereby protecting against psychological outcomes such as anxiety and depression.

### Heart-rate Variability Biofeedback

Biofeedback is a wellness-oriented intervention that aims to reduce stress by training individuals to control physiological processes such as breathing and heart rate ( Ratanasiripong *et al.*, 2010). Biofeedback promotes self-regulation and may influence affect and psycho-physiological processes. Visted and colleagues identified a link between impaired emotion regulation capacity and decreased HRV measured using biofeedback devices ( Visted *et al.*, 2017). Previous studies have used biofeedback to train college students in regulating HRV to better manage stress, anxiety, and attentional symptoms ( Ratanasiripong *et al.*, 2010). Biofeedback has been applied with practicing physicians as well; physicians who used a portable biofeedback device three times per week reported significantly reduced short-term perceived stress compared to control ( Lemaire *et al.*, 2011). Biofeedback is a wellness intervention with the potential to teach medical students to enact measurable changes in their emotion regulation.

### Structured-napping

Sleep plays an important role in emotion regulation ( Gruber and Cassoff, 2014). Inadequate sleep is associated with a host of health issues, including mood impairment and compromised learning and memory ( Killick, Banks and Liu, 2012; Hershner and Chervin, 2014; Spira *et al.*, 2014). Despite limited research, there is widespread awareness of sleep-related concerns in this population ( Azad *et al.*, 2015). Studies on countering the effects of inadequate sleep in residents may be generalizable to students, specifically the positive effects of structured napping. A recent study examined the use of brief, 20-minute naps on the neuro-cognitive performance of first-year internal medicine residents and found napping to be associated with increased cognitive alertness and fewer mistakes throughout the day ( Amin *et al.*, 2012). This finding is promising in that napping need not be lengthy to generate benefits.

Following review of the Association of American Medical Colleges (AAMC) Year Two Questionnaire (Y2Q) Individual School Report, our institution sought to expand wellness offerings for medical students. This provided an opportunity to examine whether specific wellness interventions, namely biofeedback and structured napping, would yield improvement in reducing burnout and stress and improving wellbeing of students. We used emotion regulation theory to inform our selection of biofeedback and structured napping for this study. The aims of this pilot study were to:

1. Assess engagement in wellness behaviors by medical students, and
2. Explore the effects of biofeedback and structured napping on wellness variables in medical students using pre-/post-scores on instruments measuring self-reported empathy, stress, burnout, and distress

## Methods

### Design and Setting

We conducted a randomized, controlled trial of biofeedback and structured napping use by first (M1) and second (M2) year medical students at University of Central Florida College of Medicine. Recruitment and baseline measures took place between August-September 2016, with study follow-up ending in March 2017. Approval was obtained from the University of Central Florida Institutional Review Board (IRB).

Students were informed of the study during M1 and M2 orientation weeks in August 2016. One of the investigators (KDD) gave presentations to each respective class on wellness promotion and the role of sleep and biofeedback to manage stress during medical school. We also conducted four tabling events to answer students’ questions regarding the study and demonstrate the biofeedback device. As part of the demonstration for biofeedback, brief self-guided breathing techniques were taught to participants to illustrate how to alter one’s physiological responses with guided breathing.

### Participants

All M1 (n = 121) and M2 (n= 123) students enrolled at University of Central Florida College of Medicine in August 2016 were eligible to participate in the study. The following inclusion/exclusion criteria were used: (a) age 18 or older, (b) currently enrolled as a UCF medical student (c) no known medical impairments that impact sleep (narcolepsy, sleep apnea) or cardiac conditions that impact heart rate (cardiac arrhythmia, abnormal heart rate), and (d) do not self-report being pregnant. There were no exclusions pertaining to mental health diagnoses.

### Materials

#### Biofeedback

To measure biofeedback, a portable hand-held device was used to record participants’ HRV measurement while they engaged in 3-5 minutes of self-guided breathing exercisesThe Inner Balance technology analyzes and displays our heart rhythm, measured by Heart Rate Variability (HRV), which indicates how emotional states are affecting our nervous system. The Inner Balance technology analyzes and displays our heart rhythm, measured by Heart Rate Variability (HRV), which indicates how emotional states are affecting our nervous system. The Inner Balance technology analyzes and displays our heart rhythm, measured by Heart Rate Variability (HRV), which indicates how emotional states are affecting our nervous system. The Heartmath Biofeedback® with Inner Balance® software was used, which included an adapter that attached to participants’ iPhone or iPad with a sensor that clipped to users’ earlobes. HRV-biofeedback incorporates a measure of heart inter-beat intervals in a reflection of an individual’s physiological state, i.e., vagal tone vs. sympathetic nervous system activation ( Visted *et al.*, 2017). These HRV recordings were stored in a heart-cloud database, and participants were invited to share access to their individual HRV readings with the researchers.

#### Structured-Napping/Energy Pods

To standardize the use of structured napping in this study, participants used energy pods developed by MetroNaps Energy Pods® located in the University of Central Florida College of Medicine Library. These pods are designed so that participants enter the napping “shell,” and naps are pre-set for 20-minutes. At the end of the nap, the shell vibrates and gradually lights up to wake the occupant. A general record of use during the study was maintained, but it was not linked to specific participants.

### Survey Instruments

Participants in this study completed six psychosocial self-report surveys. The surveys were intentionally selected to correspond with AAMC’s Year Two Questionnaire (Y2Q). See Table 1 for detailed survey items.

*Demographic data questionnaire*- Medical students were asked to provide demographic information including age, gender, ethnicity, year in school, relationship status, debt, sleep, and time engaged in wellness behaviors.

*Interpersonal Reactivity Index* ( *IRI*) ( Davis, 1980) - We used two of the four subscales, Perspective Taking and Empathic Concern, which were deemed most important for medical students. Items are answered on a 5-point Likert scale ranging from “Does not describe me well” to “Describes me very well.” Reliability of the IRI in this study was estimated at .82 using Cronbach’s alpha.

*Perceived Stress Scale (PSS)* ( Cohen, Kamarck and Mermelstein, 1983) - PSS is an original 10-item inventory that was adapted to 4-items (PSS-4) for this study. The questions ask about respondent’s feelings and thoughts in the last month. Items are scored on a 5-point Likert scale ranging from 0-Never to 4-Very often. Higher scores on this measure indicate higher perceived stress. Reliability estimates for the PSS are consistently measured at greater than .70 ( Lee, 2012).

*Quality of life scale (QOL)* - QOL was measured using the LASA-6 which consist of six single items asking respondents to rate, on a zero to ten scale, their perceived level of functioning. Items asked about overall quality of life, intellectual, physical, emotional and spiritual well-being, as well as level of social activity. Higher scores indicate greater quality of life. Reliability of the QOL in this study was estimated at .83 using Cronbach’s alpha.

*Oldenburg Burnout Inventory (OLBI-MS) *( Halbesleben, *et al*., 2005) - The OLBI is a tool used for the assessment of burnout. The OLBI is a 16-item scale that measures burnout and disengagement on a 4-point scale ranging from 1 (Strongly agree) to 4 (Strongly disagree). Higher scores indicate higher levels of burnout. Reliability of the OLBI in this study was estimated at .78 using Cronbach’s alpha.

*Physician Well-Being Index* *(PWBI) (Dyrbye et al., 2013)* - The PWBI is a brief 7-item screening tool to identify physicians in distress. Items are scored with either a yes (1) or no (0). Questions assess burnout, fatigue, anxiety, depression, feeling overwhelmed, and inability to carry out personal responsibilities. Higher scores indicate higher levels of distress. A score of 5 or greater is indicative of poorer mental health and well-being. Reliability of the PWBI in this study was estimated at .72 using Cronbach’s alpha.

**Table 1.** Questionnaire Items

Interpersonal Reactivity Index (IRI)
1. I sometimes find it difficult to see things from the “other guy’s” point of view. (PT) (-)
2. I try to look at everybody’s side of a disagreement before I make a decision. (PT)
3. I sometimes try to understand my friends better by imagining how things look from their perspective. (PT)
4. If I’m sure I’m right about something, I don’t waste much time listening to other people’s arguments. (PT) (-)
5. I believe that there are two sides to every question and try to look at them both. (PT)
6. When I’m upset at someone, I usually try to “put myself in his shoes” for a while. (PT)
7. Before criticizing somebody, I try to imagine how I would feel if I were in their place. (PT)
8. I often have tender, concerned feelings for people less fortunate than me. (EC)
9. Sometimes I don’t feel very sorry for other people when they are having problems. (EC) (-)
10. When I see someone being taken advantage of, I feel kind of protective towards them. (EC)
11. Other people’s misfortunes do not usually disturb me a great deal. (EC) (-)
12. When I see someone being treated unfairly, I sometimes don’t feel very much pity for them. (EC) (-)
13. I am often quite touched by things that I see happen. (EC)
14. I would describe myself as a pretty soft-hearted person. (EC)
**Perceived Stress Scale (PSS)**
1. In the last month, how often have you felt that you were unable to control the important things in your life?
2. In the last month, how often have you felt confident about your ability to handle your personal problems?
3. In the last month, how often have you felt that things were going your way?
4. In the last month, how often have you felt difficulties were piling up so high that you could not overcome them?
**Quality of life scale (QOL)**
1. Your overall quality of life?
2. Your overall mental (intellectual) well-being?
3. Your overall physical well-being?
4. Your overall emotional well-being?
5. Your level of social activity?
6. Your spiritual well-being?
**Oldenburg Burnout Inventory (OLBI-MS)**
1. I always find new and interesting aspects in my work.
2. There are days when I feel tired before I arrive at work.
3. It happens more and more often that I talk about my work in a negative way.
4. After work, I tend to need more time than in the past in order to relax and feel better.
5. I can tolerate the pressure of my work very well.
6. Lately, I tend to think less at work and do my job almost mechanically.
7. I find my work to be a positive challenge.
8. During my work, I often feel emotionally drained.
9. Over time, one can become disconnected from this type of work.
10. After working, I have enough energy for my leisure activities.
11. Sometimes I feel sickened by my work tasks.
12. After my work, I usually feel worn out and weary.
13. This is the only type of work that I can imagine myself doing.
14. Usually, I can manage the amount of my work well.
15. I feel more and more engaged in my work.
16. When I work, I usually feel energized.
**Physician Well-Being Index (PWBI)**
1. Have you felt burned out from your work?
2. Have you worried that your work is hardening you emotionally?
3. Have you often been bothered by feeling down, depressed, or hopeless?
4. Have you fallen asleep while stopped in traffic or driving?
5. Have you felt that all the things you had to do were piling up so high that you could not overcome them?
6. Have you been bothered by emotional problems (such as feeling anxious, depressed, or irritable)?
7. Has your physical health interfered with your ability to do your daily work at home and/or away from home?

#### Procedure

Study enrollment began during the first two weeks of the 2016-2017 academic semester. Three recruitment methods were used including a presentation during orientation, email and social media advertisement, and tabling events. Interested participants who met inclusion criteria completed an online survey link of baseline measurements and a baseline HRV reading. An incentive of up to $25 was offered to participants.

Forty-two students completed baseline data and were selected for the final study (See Table 2). Students were randomly assigned to one of three conditions: control (“wellness as usual,” n=10), biofeedback (n=16), or structured napping (n=16). Students were instructed to use the biofeedback device for 3-5 minutes three times per week, and students in the napping condition were instructed to use the napping pods three times per week for 20 minutes per nap. After Time 2 data collection, which occurred approximately 10 weeks into the 20-week study, participants in all conditions were permitted to use any wellness modality (napping, biofeedback, or others), and use of these wellness activities was measured by self-report. The authors opted to not restrict engagement in wellness activities after Time 2 (including allowing participants to engage freely in either structured napping or biofeedback). This decision was influenced by the length of the study and interest in not only comparing biofeedback and structured napping but examining the impact of combined wellness overall. Time 3 data was collected at the semester end. Compliance for biofeedback was tracked by participants sharing HRV readings with the researchers. For structured napping, a general record of napping pod usage was monitored during the study period.

**Table 2.** Demographic characteristics of Study Participants

Demographic Characteristics	n=42
Gender	
Male	15
Female	26
Non- reporting/responding	1
Age, Median	24
Race/Ethnicity	
White, non-Hispanic/Latino	32
Asian American	9
African American	0
Hispanic/Hispanic American/Latino(a)	1
Other	0
Relationship Status	
Single	32
Married	4
Divorced	0
Partner	5
Other	1
Year in Medical School	
M-1	9
M-2	33
Current level of financial debt (dollars)	
< $50,000	15
$50,000 - $100,000	19
> $100,000	8

## Results/Analysis

Data was analyzed using the IBM SPSS statistics software package. With regard to demographics, 76% of this sample identified as non-Hispanic White American (n=32); 21% as Asian American (n=9), and 2% (n=1) as Hispanic. Twenty-six of the participants in this study were female, 15 were male, and one preferred not to specify gender.

During the course of the study, participants recorded 276 structured naps and 24 biofeedback recordings. As part of this study, we were also interested in obtaining assessment of baseline engagement in wellness behaviors. We asked about mindfulness, exercise, use of peer support, sleep and napping behaviors, and time with an animal (Refer to Table 3). Similar to other studies done with medical students and residents, participants in this sample reported less than seven hours of sleep per night (M=6.8; SD=.86). We found that engagement in specific wellness activities at Time 1 was associated with engagement in other wellness activities at later time points. For example, use of the energy pods was positively correlated with mindfulness meditation (r=.424, n=32, p<.05) and exercise using walking treadmills provided by the school (r=.589, n=32, p<.05).

**Table 3.** Average hours of self-reported engagement in wellness activities at baseline

**Participation in wellness activities** (hours per week over the previous month)	Mean	SD	N
Energy/sleep pods	.33	.55	33
Exercise (running, swimming, aerobics, other exercise class)	7.64	11.69	40
Mindfulness meditation	.32	.58	32
Peer support program (PSP)	.00	.00	31
Time with animal/pet	11.11	18.95	35
Walking treadmills	3.23	5.63	33
Hours of sleep, average per night	6.80	.86	42

We hypothesized that use of structured napping and biofeedback would be associated with increased empathy (Interpersonal Reactivity Index), decreased stress (Perceived Stress Scale), reduced burnout (Oldenburg Burnout Inventory), and reduced distress (Physician Well-Being Index). The hypothesis was partially supported. Self-reported hours of sleep at T1 was positively correlated with item 2 on the Perceived Stress Scale at T3, “In the last month, how often have you felt confident about your ability to handle your personal problems?” (r=.422, n=17 p<.05). This indicates that more sleep at the start of the study is associated with increased confidence in managing personal stress at the end of the study period. Self-reported sleep at T1 was negatively correlated with item 4 on the Perceived Stress Scale at T3, “In the last month, how often have you felt your difficulties were piling up so high that you could not overcome them?” (r=-.475, n=17, p<.05). This finding indicates that more sleep at the start of the study is a protective factor in managing stress over time.

Participants assigned to either of the intervention groups (biofeedback or energy pods) experienced less distress as measured by Dyrbye’s Physician Well-Being Index (Dyrbye *et al.*, 2013) compared to those initially assigned to the control group from Time 1 to Time 3. We measured this by first calculating the rate of self-reported distress at Time 1 to Time 3. Twenty-one percent of participants in the entire sample met the criteria for clinical distress at Time 1 compared to just 11% of the sample at Time 3. We noted differential attrition with a higher proportion of participants in the intervention groups continuing with the study. An independent samples t-test was used to examine possible group differences in well-being at Time 3 between intervention (biofeedback and energy pod combined; M=2.46, SD=1.81) and control group (M=3.75, SD=.96) and the finding was approaching significance (t=1.86 (15), p=.092). Refer to Tables 4 and 5 for detailed results.

**Table 4.** Between Group Comparisons of Participants Assigned to Control Compared to those Assigned to One of the Interventions (Biofeedback or Structured Napping) from T1 to T3

Outcome Measure	Time 1		*t*	Time 3		*t*
	*Control (10)*	*Intervention (30)*		*Control* *(4)*	*Intervention* *(13)*	
Interpersonal Reactivity Index (IRI)	M=38.9 SD=7.06	M=40.1 SD=5.95	-.527	M=39.25 SD=1.71	M=40.77 SD=5.18	-.566
Perceived Stress Scale (PSS)	M=9.00 SD=1.49	M=9.41 SD=1.23	-.889	M=8.50 SD=1.29	M=9.31 SD=.95	-1.378
Quality of life scale (QOL)	M=40.40 SD=4.95	M=41.19 SD=8.58	-.277	M=43.50 SD=6.25	M=39.54 SD=6.79	1.037
Oldenburg Burnout Inventory (OLBI-MS)	M=32.1 SD=3.96	M=31.23 SD=4.85	.515	M=30.75 SD=2.99	M=30.85 SD=4.32	-.041
Physician Well-Being Index (PWBI)	M=3.70 SD=1.83	M=2.71 SD=1.90	1.445	M=3.75 SD=.957	M=2.46 SD=1.81	1.858 *

** indicates significant at the .05 level

* indicates significant at the .01 level

**Table 5.** Between Group Comparisons of Participants Assigned to Biofeedback Compared to those Assigned to Structured Napping from T1 to T3

Outcome Measure	Time 1		*t*	Time 3		*t*
	*Biofeedback* *(15)*	*Structured Nap (16)*		*Biofeedback* *(5)*	*Structured Nap* *(8)*	
Interpersonal Reactivity Index (IRI)	M=39.71 SD=5.65	M=40.44 SD=6.37	-.327	M=39.60 SD=1.67	M=41.50 SD=6.55	-.627
Perceived Stress Scale (PSS)	M=9.27 SD=1.39	M=9.56 SD=1.09	-.662	M=9.40 SD=.548	M=9.25 SD=1.17	.267
Quality of life scale (QOL)	M=42.33 SD=9.68	M=40.13 SD=7.56	.710	M=37.00 SD=5.29	M=41.12 SD=7.45	-1.072
Oldenburg Burnout Inventory (OLBI-MS)	M=31.33 SD=5.11	M=31.12 SD=4.77	.117	M=33.60 SD=3.21	M=29.13 SD=4.16	2.045 *
Physician Well-Being Index (PWBI)	M=2.13 SD=1.68	M=3.25 SD=1.98	-1.68	M=3.00 SD=1.73	M=2.13 SD=1.89	.838

** indicates significant at the .05 level

* indicates significant at the .01 level

Biofeedback data was only available for six participants; thus it was not able to be analyzed statistically. Participants had the option to provide a code for the research team to access their biofeedback recordings, and not all participants chose to share their readings. We calculated means for heart rate, coherence, and achievement for the first (measured at Time 1) and last available biofeedback reading (measured during Time 3) of the six participants who did consent to sharing their recordings. The mean heart rate for Time 1 was 70.33 (SD=13.78) compared to 64.25 (SD=5.68) at Time 3. Means for coherence increased from Time 1 (M=3.32; SD=1.48) to Time 3 (M=4.23; SD=1.76), as did means for achievement from Time 1 (M=143.50; SD=67.02) to Time 3 (M=281.75; SD=85.62). However, due to the small n, we are unable to determine if these changes are statistically significant.

## Discussion

Efforts by medical schools to promote student well-being and mitigate stress will need to encompass not only support for students’ self-initiated efforts, but also institution-initiated programs ( Slavin, Schindler and Chibnall, 2014). Institution-initiated programs serve as an endorsement for promotion of wellness activities and are essential to creating a learning climate that reduces stigma and barriers to accessing these resources ( Dyrbye, et al., 2012, Slavin et al., 2014). Institution-initiated mind-body programs can provide structured opportunities to improve students’ emotion regulation skills and adaptive response to stress. Furthermore, students’ ability to use adaptive emotional regulation strategies may mitigate the development of psychological symptoms.

In this pilot study we sought to gain a baseline assessment of medical students’ engagement in wellness behaviors and to evaluate the effectiveness of two institution-initiated interventions, namely biofeedback and structured napping on measures of stress and burnout.

The findings of this pilot study suggest that students who engage in self-initiated wellness behaviors are more likely to adopt new wellness activities, so it may be important to introduce and promote these behaviors early in the course of professional education.

Of our two interventions, the structured napping generated more promising results, particularly the association with reduced stress scores. This is consistent with the findings of Amin and colleagues ( Amin *et al*., 2012) regarding the improvements in neurocognitive function associated with a mid-day nap in a group of residents. Although we hypothesized the anticipated protective effects of biofeedback, we did not achieve sufficient study engagement in this intervention to draw any conclusions. We were also unable to demonstrate changes to empathy scores due to the small sample size.

The effectiveness of the napping pods can be attributed to several factors. Students who utilized these may have perceived an immediate benefit, encouraging their continued use. The napping pods were located in an alcove off the entrance to the library, granting a frequent visual reminder of their availability. In contrast, students wishing to use biofeedback were required to check out a HRV earlobe sensor from the library and had to take extra steps online to share their data with the research team. This created obstacles to the ease of use of the biofeedback and our ability to capture user data. Further exploration of biofeedback is warranted by making the devices readily available to students and using both quantitative and qualitative approaches.

Our study has several limitations. The small sample size limits our ability to offer conclusions. The reliance on students to share their biofeedback use data likely led to underreporting of use. Furthermore, the napping pods did not have a feature for tracking individual participants’ frequency and duration of naps. Future studies would benefit from incorporating a personalized app (e.g. Fitbit®) to allow for more accurate tracking of these wellness interventions. Selection bias may have also influenced our findings, in that participants who chose to enroll in this study may have an increased interest for engagement in wellness. Since the follow-up period was limited to 20 weeks, we cannot conclude that these benefits are sustained over longer periods. Finally, although we selected a *conceptual* framework, namely emotion regulation, to inform the selected wellness interventions of biofeedback and structured napping, future studies will benefit from obtaining empirical data to confirm the association between emotional regulation and the selected interventions.

## Conclusion

In conclusion, medical students are engaging in a variety of wellness behaviors; medical schools should continue to promote sleep, mindfulness, and exercise to help students manage stress. Future studies should assess wellness activities for all medical students to provide data that informs judicious allocation of resources for wellness interventions. Structured napping is promising for reducing stress and improving well-being. Biofeedback results were inconclusive in this study, and more data is needed. Due to limited sample size and loss of data, we were unable to assess the impact of biofeedback and napping on emotion regulation of participants, though this is an important area for future research.

Although a pilot, our study examined the potential role of two institution-initiated strategies for promoting student wellness, and we used emotional regulation as a conceptual framework informing our selection of interventions. We hope this will lead to further exploration of the optimal role for these approaches as part of programmatic offerings to medical students to promote positive mental health and reduce burnout during medical school and beyond.

## Take Home Messages

• Student well-being efforts should include encompass both student self-initiated and institution-initiated programs.
• It is important to introduce and promote well-being activities early in the course of professional education.
• Structured napping warrants further study as a strategy for stress reduction.

## Notes On Contributors

Dr. Hernandez is Associate Professor, Departments of Internal Medicine and Medical Education, University of Central Florida, Orlando, FL.

Dr. Daly is Director of Counseling & Wellness Services and Assistant Professor, Department of Clinical Sciences, University of Central Florida, Orlando, FL.

Dr. Mehta is Assistant Professor, Department of Clinical Sciences, University of Central Florida, Orlando, FL.

Dr. Verduin is Professor and Associate Dean for Students, Departments of Medical Education and Clinical Sciences, University of Central Florida, Orlando, FL.
