# Supplementary material for: A Pilot Study Examining Biofeedback and Structured Napping to Promote Medical Student Wellbeing
Source: MedEdPublish (2016). 2019 May 27;8:110. [Version 1] doi: 10.15694/mep.2019.000110.1 (PMC10712478; doi:10.15694/mep.2019.000110.1)
Supplement: Peer review history

# Reviewer report: Reviewer response for version 1

**DOI:** 10.21956/mep.19825.r29866

**Published:** 2019-08-20

**Authors:** Ken Masters

**Affiliations:** 1 Sultan Qaboos University

This review has been migrated. The reviewer awarded 4 stars out of 5 An interesting, and very well-written pilot study examining biofeedback and structured napping to promote medical student wellbeing. Overall, the entire study has been thoroughly conducted and written up. There are, however, just a few small details that need to be addressed.:• I’m not sure why some significance was tested at the .05 level, and some at .01. I understand that the .01 makes it “more” significant, but, unless there is a specific reason (which should then be explained), the levels should be identical throughout.• Related to this, Tables 4 and 5 give an indication that those values with significance at .05 are indicated with the double asterisk (**). When I look at those tables, though, I do not see any indicators inside the table (that is, I do not see any numbers indicated with the double asterisk).• “We were also unable to demonstrate changes to empathy scores due to the small sample size.” (With the Interpersonal Reactivity Index). As far as I can see, you cannot really assume that the failure to find changes in the empathy scores is due to sample size. It may be that indeed, the intervention does not lead to changes in empathy scores, irrespective of sample size. So, I think that, at this stage, you can only say “We were also unable to demonstrate changes to empathy scores” and leave it at that.• A further limitation of the study is this: the existence and availability of pods. Although it is not a limitation of the study per se, it will limit the generalisability. Just as any study that uses expensive or rare technology, this does need to be acknowledged as a limitation.Minor issues • “our institution sought to expand wellness” It would be better to state the name of the institution at this point, rather than require the reader to scroll up to the top of the paper to determine this.• “Data was ” should be “Data were ”So, a really useful study, but some details need to be addressed.

Reviewer Expertise:

NA

No decision status is available

# Reviewer report: Reviewer response for version 1

**DOI:** 10.21956/mep.19825.r29867

**Published:** 2019-06-15

**Authors:** sathyanarayanan varadarajan

**Affiliations:** 1 SRM Medical College, SRM University

This review has been migrated. The reviewer awarded 5 stars out of 5 In this fascinating pilot study, the authors assessed engagement in wellness behaviors by first and second year medical students and explored the effects of biofeedback and structured napping on wellness variables in them using pre-/post-scores on instruments measuring self-reported empathy, stress, burnout, and distress by conducting a randomized, controlled trial at University of Central Florida College of Medicine. Approval was obtained from the IRB and participants were selected by using appropriate Inclusion/exclusion criteria. Forty-two students completed baseline data and were selected for the final study. Students were randomly assigned to one of three conditions: control (“wellness as usual,” n=10), biofeedback (n=16), or structured napping (n=16). Biofeedback and structured napping were measured with appropriate software.The findings of this pilot study suggest that students who engage in self-initiated wellness behaviors are more likely to adopt new wellness activities. Another finding is that the structured napping generated more promising results, particularly the association with reduced stress scores.The authors also mentioned the limitations of the study like the small sample size and the possibility of selection bias.The take home message is that, “It is important to introduce and promote well-being activities early in the course of professional education”.This study is an important contribution in the field of medical education, can help medical educators to promote sleep, mindfulness and well being activities among medical students for managing stress.

Reviewer Expertise:

NA

No decision status is available

# Reviewer report: Reviewer response for version 1

**DOI:** 10.21956/mep.19825.r29865

**Published:** 2019-06-05

**Authors:** Christine Kauffman

**Affiliations:** 1 University of Central Florida College of Medicine

This review has been migrated. The reviewer awarded 4 stars out of 5 Interesting pilot study

Reviewer Expertise:

NA

No decision status is available
